# Listening and learning: a qualitative study of Scottish care home staff experiences of managing COVID-19 between March 2020-August 2022

**DOI:** 10.1186/s12877-023-04251-z

**Published:** 2023-09-07

**Authors:** Jennifer Kirsty Burton, Maria Drummond, Katie I Gallacher, Terence J Quinn

**Affiliations:** 1 Academic Geriatric Medicine, School of Cardiovascular & Metabolic Health, College of Medicine, Veterinary & Life Sciences, University of Glasgow, Glasgow, G12 8TA Scotland; 2 Nursing & Health Care, School of Medicine, Dentistry & Nursing, College of Medicine, Veterinary & Life Sciences, University of Glasgow, Glasgow, G12 8LW Scotland; 3 General Practice & Primary Care, School of Health and Wellbeing, College of Medicine, Veterinary & Life Sciences, University of Glasgow, Glasgow, G12 8TB Scotland; 4 School of Cardiovascular & Metabolic Health, New Lister Building, University of Glasgow, Glasgow Royal Infirmary, Room 2.42 Level 2, Alexandra Parade, G31 2ER Glasgow, Scotland

**Keywords:** Care homes, Long-term care, COVID-19, Relationships, Adaptation, Inequalities, Psychological impacts, Compassionate leadership, Positive support, Involvement

## Abstract

**Background:**

The serious outcomes of outbreaks of COVID-19 in care homes have been described internationally. The experiences of professionals working through outbreaks has received less attention, missing opportunities to acknowledge and learn lessons. Our aim was to explore the experiences of care home staff in Scotland of managing COVID-19 within their homes to help inform understanding and future practice.

**Methods:**

From April to August 2022, 34 individual semi-structured interviews were conducted with care home staff working in homes which experienced an outbreak(s) of COVID-19. Reflexive thematic methods were used to analyse verbatim deidentified transcripts.

**Findings:**

There was no singular experience of COVID-19 outbreaks within care homes. We identified four broad groupings of homes with outbreaks (significant outbreaks, managed outbreaks, outbreaks in remote/rural homes & outbreaks in homes supporting younger adults), with overlaps in timing and severity and variation in the support received and impact. The national response to the COVID-19 pandemic resulted in fundamental change to care home relationships. Staff responded by adaptation in uncertainty. However, they were challenged by emerging inequalities influencing residents’ care. There were tensions between staff experience and evolving external approaches to regulation and oversight. All this change resulted in psychological impacts on staff. However, there was also widespread evidence of compassionate leadership and teamwork in their responses. Effective sources of support were underpinned by respectful relationships and continuity, tailored to individual contexts.

**Conclusions:**

The lived experiences of care home staff during the COVID-19 pandemic provide valuable insights applicable beyond the pandemic context. This includes: recognition of the specialism, complexity and diversity of care home practice; the value afforded by embedding genuine representation and involvement in planning, policy-making and research; the need for individualising to people in their contexts and the value of fostering respectful relationships across professional groups to support residents.

**Supplementary Information:**

The online version contains supplementary material available at 10.1186/s12877-023-04251-z.

## Background

The disproportionate negative impacts of the COVID-19 pandemic on adults living in long-term care settings are well-documented. While early analyses focused on epidemiological data, [1, 2] more recently research has sought to learn from those with professional experience, [3, 4] and to evaluate international policy differences in responses [5, 6]. This work has included a range of methodologies including surveys, [7, 8] in-depth interviews [9] and focus groups [10] seeking to collate staff experiences. Some have focused on specific professional groupings, considering the impact on registered nursing staff [9, 11] or care home managers [12–14] and how the pandemic impacted their specific roles. The interconnectedness and interdependencies of care homes within their communities is increasingly evident. However, despite the growth of international research Jones et al. described important gaps in their 2022 scoping review including learning from staff experiences to ensure better pandemic preparedness and support for everyday working in the sector [15].

A third of Scottish care homes experienced an outbreak during the first wave of infections, most outbreaks occurred in older adult services [16]. There was significant heterogeneity in outbreak size, duration and fatality with a concentration of mortality among a small number of homes [17]. Qualitative research undertaken in Scotland has focused on the earliest phase of the pandemic, considering adaptations by staff in enabling communication between residents and families [18] and evaluating the impact of visiting restrictions [19]. One care provider collaborated in a study of stress and coping, concluding a strategic approach to psychological support is required for the workforce [20].

Health and social care are devolved issues within Scotland. Consequently, there have been differences in practice, policy and political responses compared to the rest of the United Kingdom (UK), some of which are the subject of a Public Inquiry (**Supplementary file 1**) [21]. National bodies have undertaken evaluations of the pandemic response, including the impact on care homes [22–24]. However, the voice and experiences of staff have not been heard, missing opportunities to learn.

Our aim was to explore the experiences of care home staff in Scotland of managing COVID-19 to help inform understanding and future practice.

## Methods

This study has been reported in accordance with the Consolidated criteria for Reporting Qualitative research checklist [25].

### Setting

Care homes in Scotland are defined as: 24-hour residential care facilities, some have on-site registered nursing staff, all are registered by the Care Inspectorate (*national scrutiny body and care regulator for all care services*) [26]. Three quarters are for older adults, with the remainder supporting those living with learning disabilities, mental health problems, physical and sensory impairments and substance misuse [27].

### Research team and reflexivity

Data collection was undertaken by two female clinical academics. JKB is a Registrar in Geriatric Medicine and postdoctoral clinical lecturer who worked in acute and geriatric medicine inpatient services during the pandemic. MD is a postdoctoral nurse academic who worked as a District Nurse during the pandemic, with prior experience working in care homes. Both had experience undertaking interviews during doctoral research. A Study Steering Committee comprising three experienced professionals from the social care sector supported in terms of recruitment, oversight and interpretation.

All participants were informed of the purpose of the study. No additional explanation was provided around the researchers’ motivations. Five (14.7% of the sample) were known to the researchers in a professional context, working in care home research, rather than as clinicians.

### Study design

#### Theoretical framework

Reflexive thematic analysis was undertaken [28]. This approach suited the experiential nature of the data, allowing analysis that generated insights of shared meaning [29]. Analysis was undertaken inductively, although prior interest in the topic will have resulted in some deductive reasoning (for example exploring access to testing). Themes were developed from the data, focusing on semantic insights [30]. The analysis was approached from a critical realist perspective, hearing participants’ perceptions of their diverse lived experiences during the pandemic, influenced by their context of practice. We brought these accounts together, providing interpretation of the dataset as a whole, supported by their accounts, recognising that differences in experience were influenced by both individual characteristics (such as role, experience) and context of practice (type of home, size of outbreak, timing etc.) [28].

#### Participant selection

We sought a diverse sample of participants working in adult care home services, across urban and rural areas. We invited individuals with direct experience of a COVID-19 outbreak in their service (involving residents, staff or typically both groups).

Recruitment, through posters and brief explanatory text, was facilitated by professional networks, Enabling Research in Care Homes (ENRICH) Scotland, Scottish Care and National Health Service (NHS) and care home stakeholders participating in the Scottish Government Clinical and Professional Advisory Group for Adult Social Care (CPAG) group.

A total of 59 individuals expressed interest and 57 were sent the study documents. Two were ineligible to participate as they had not been directly employed in care homes. A total of 37 individuals replied, 34 were recruited and interviewed. Of the three non-participants: one dropped-out due to ill health, one did not attend an interview and one did not arrange an interview.

#### Setting

Interviews were undertaken remotely by phone or video-call (based on participant preference). Most were interviewed in their workplace, some at home. All were encouraged to find a quiet place and generally participants were alone. Occasionally individuals were in shared offices and some participants confirmed information with colleagues. Several interviews were temporarily paused to allow participants to respond to urgent work issues.

#### Sample characteristics

Due to the sensitivity of the topic, we purposively did not collect care home identifiers and de-identified care home names, regions and individuals. We did not collect demographic information. Instead, we asked participants to define their role in the care home, duration of time working in adult social care (not limited to current role), working patterns, services covered during the pandemic, service type (older adult/other adult), broad geography (urban/rural) and size (number of beds).

#### Data collection

A semi-structured interview guide was developed, with input provided from care home representatives and other interested stakeholders via the CPAG group (**Supplementary file 2**). Minor revisions were made following initial interviews. All participants were interviewed once. Audio recordings were transcribed verbatim by a transcription service. Transcripts were checked for accuracy and deidentified by the researchers. Researchers made notes during and at the end of interviews. Data saturation was not sought [31] and recruitment ended when all those wishing to participate had been interviewed.

### Analysis

Reflexive analysis[24] was undertaken, generating themes from the data:

#### Familiarisation

JKB and MD listened to the audio and read and re-read transcripts independently making notes to add to their personal reflections at the time of interviews.

#### Coding

they met in person reviewing a printed transcript, generating and mapping codes using index cards, highlighting extracts of text. Codes were discussed and debated, challenging assumptions made from professional or personal experiences. A second stage of coding was undertaken by JKB using NVivo software (*version 12*) with transcripts approached in a different sequence to re-examine codes and ensure coding had not been influenced by examples in early or particularly emotive interviews.

#### Generating initial themes

codes were collated into potential themes by reviewing the extracted data and creating visual mind-maps of similar and distinct concepts to allow flexibility in organisation and explore relationships between themes.

#### Developing and reviewing themes

data were examined to ensure themes reflected both selected extracts and the whole dataset. This was approached iteratively with questioning and discussion with senior team members to help define the relationship between themes.

#### Refining, defining and naming themes

proposed themes were then shared with the wider team and with the Study Steering Group. This discussion helped in improving naming of themes to ensure shared meaning and clarity around the relationships between themes.

## Results

### Participants and settings of practice

Our sample are social care professionals working in a range of roles and settings (Table 1).

**Table 1:** Characteristics of study participants

Category	Responses	Number of participants (%)
**Role(s) of participant** ^**1**^	Manager, Nursing background Manager Senior Management, Nursing background Carer/Senior Carer Deputy Manager, Nursing background Advanced Nurse Practitioner Agency Nurse Owner & Manager Senior Management Senior Management, Allied Health Professional	11 (32.4) 6 (17.6) 6 (17.6) 3 (8.8) 3 (8.8) 1 (2.9) 1 (2.9) 1 (2.9) 1 (2.9) 1 (2.9)
**Years working in adult social care**	One to five Six to nine Ten to fifteen Sixteen to twenty More than twenty	4 (11.8) 2 (5.9) 7 (20.6) 5 (14.7) 16 (47.1)
**Working pattern**	Full-time Part-time	32 (94.1) 2 (5.9)
**Experience during the pandemic**	One care home service More than one care home service Care homes and NHS services	18 (52.9) 12 (35.3) 4 (11.8)
**Type of care home service**	Older adult service Other adult service Both service types	28 (82.4) 4 (11.8) 2 (5.9)
**Geography of care home service**	Urban Rural Both geographies	23 (67.6) 6 (17.6) 5 (14.7)
**Number of beds** **(Care home size)**	Less than 30 beds *(small)* 31 to 60 beds *(medium)* 61 to 90 beds *(large)* More than 90 beds *(very large)* Multiple sizes	12 (35.3) 12 (35.3) 3 (8.8) 3 (8.8) 4 (11.8)

Footnotes: 1. Nursing participants also classified themselves as general adult or mental health trained, sometimes with qualifications in both. Due to small numbers, we have not further sub-classified participants by nursing background

**Fig. 1:**
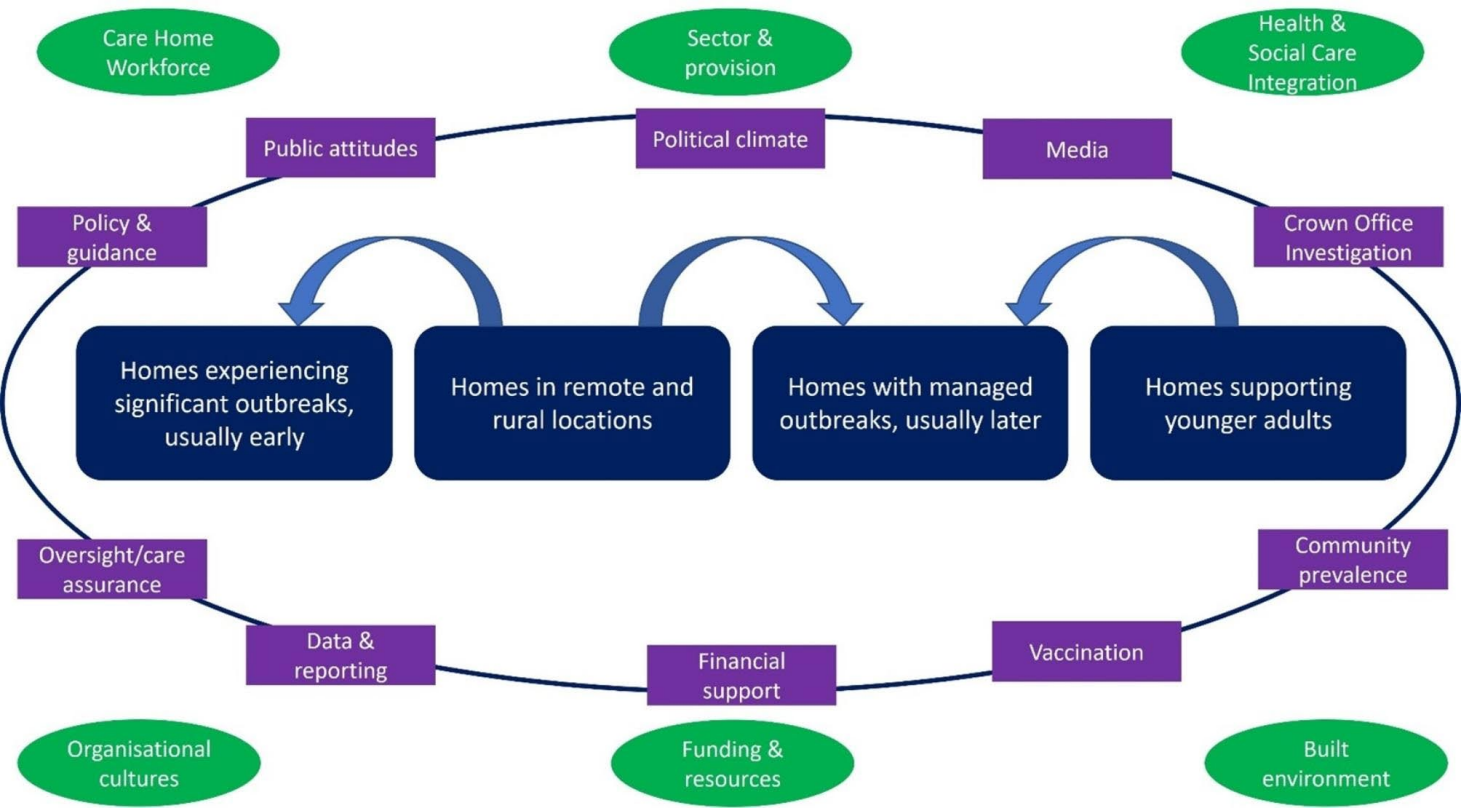
Overview of the care home outbreak experiences with established and evolving contextual factors during the pandemic in Scotland. Green ovals – established practice context; Purple rectangles – evolving context during course of the pandemic. **Crown Office Investigation**– established in May 2020 and required reporting of all care home onset deaths from COVID-19 deaths until December 2022 for investigation by Police Scotland, in an investigation known as Operation Koper. **Oversight/care assurance –** multi-protection clinical oversight teams established in each Health Board area in May 2020. **Vaccination** – older adult care home residents were prioritised for vaccination and the programme commenced in December 2020. **Community prevalence** – after the initial national lockdown (23/3/20 to 19/6/20), Scotland applied different levels of restrictions based on local community prevalence data

Interviews lasted between 31 and 70 min (mean 54). There is no singular experience of COVID-19 outbreaks within care homes. We identified four broad groupings of homes with outbreaks (significant outbreaks, managed outbreaks, outbreaks in remote/rural homes & outbreaks in homes supporting younger adults), with overlaps in timing and severity. This delineation is important to acknowledge explicitly as the care home experience of COVID-19 is often presented in public discussion as homogenous, with a focus on early serious outbreaks in older peoples’ homes. In contrast, our analysis found differential impacts based on timing, control, support, resident population and geography which are important to describe to reflect the diversity of adult care home services experiences of COVID-19 outbreaks in Scotland. In addition, the context in which homes were operating before the pandemic and evolving contextual factors which influenced experiences are visualised in Fig. 1.

### Overview of themes

The national response to the COVID-19 pandemic resulted in **fundamental change to care home relationships**. Staff responded by **adaptation in uncertainty**. However, they were challenged by **emerging inequalities influencing residents’ care**. National bodies (regulator, government, and national public health) roles and involvement in care homes changed significantly in terms of issuing guidance, changing inspection arrangements, and introducing oversight structures. There were **tensions between staff experience and these evolving external approaches**. All this change resulted in **psychological impacts** on staff. However, there was also widespread evidence of **compassionate leadership and teamwork** in their responses. Each theme will be examined below with additional illustrative quotations in **Supplementary file 3**.

### Fundamental change to care home relationships

Care home communities are varied and personal, with many residents having strong connections inside and outside the home. These relationships were altered by the national response and by care home guidance:

“It was no longer a social place or a place to have this joy, this, I don’t know, sense of community, sense of belonging, sense of being with others and we had this stillness but not a pleasant stillness, not a mindful stillness. It was more a kind of, yes, there’s something missing here. It was the heart missing from it.” *(24, Senior management & nurse, older adult homes)*.

Restricted use of communal spaces was challenging, as it was seen as an environment to foster relationships. Physical distancing requirements affected staff’s ability to support each other.

#### Visiting restrictions

Visiting restrictions were acknowledged to have been difficult for both residents and those who care for them:

“See if you could bottle the essence of what is achieved by a resident…. What they get from the interaction with their families, if you could get the essence of that and bottle it and make it into a medicine that could be formed into a tablet? Give it to everybody in the world and the world would be a better place” *(3, Senior carer, older adult home)*.

There was a collective sense that maintaining relationships was given lower priority in decision-making by those in authority than staff felt appropriate. They saw detrimental impacts on mood, behaviour, engagement and cognition from prolonged isolation. Several participants gave examples of residents for whom they felt this had contributed to their deaths. There was universal support for new approaches enabling visiting during outbreaks [32] recognising that blanket prolonged bans had been harmful. Variation in managing visiting across the sector had resulted in distress and conflict, and there was recognition of the importance of open communication.

Restrictions resulted in particularly disrupted relationships for those newly moved-in due to a ban on visits prior to moving in, initial isolation periods, PPE use and social distancing resulting in a lack of familiarity with staff and other residents. Services for younger adults sometimes chose to defer new moves or adapt guidance to accommodate specialist needs. Disruption to respite and day care affected relationships, with homes in rural areas particularly convinced of the mutual benefits to residents and their community peers.

#### End of life care

End-of-life care was recognised to be an important aspect of relational care home practice, one where staff had previously felt skilled and supported. Death and dying were radically different due to the virus itself (often resulting in unpredictable and rapid deterioration) and impact of restrictions:

“even from everything I’ve looked at about frailty, it wasn’t even frail people, you just didn’t know you literally didn’t know…if you heard somebody had it, it wasn’t like you knew what care to give them to give them good care in their end of days. Some people, you just expected them to be okay, and they weren’t, and then there were some people who had a really awful time and came out the other side, and you didn’t know” *(30, Advanced Nurse Practitioner, older adult home)*.

Participants consistently emphasised striving to ensure that no one died alone but not having usual involvement from family members, either due to suddenness of deaths, risks to them or restrictions, created challenges:

“to be sitting in that room, holding her hand, wearing a pair of blue rubber gloves and her daughter was on my phone on FaceTime with me, it’s surreal. You sit here and you think about it and you can’t believe that we would’ve been in the state where I’m doing that. That’s a really special time. Usually when families are in the home, you’re there but you’re that stepped back because you’re allowing people to have that time….And I felt a wee bit guilty ’cause I was sitting there and her daughter wasn’t.” (6, Manager & nurse, older adult home).

While some staff were able to enable visiting at the end-of-life, there was a collective sense of regret around missed opportunities and reflections on the lasting impacts for those who were not there.

### Adaptation in uncertainty

The pandemic brought a sense of uncertainty and staff had continually had to adapt their practice. This was emphasised by those in managerial roles who questioned their abilities and reported pressure to respond and support everyone:

“there was a lot of times as a manager I felt, you know, completely out of my depth, to the point where I was thinking, you can’t do this anymore, because you’re leading something that you and nobody really knows anything about.” *(16, Manager, older adult homes)*.

The experiences of managing outbreaks were diverse based on timing, severity and impact. Significant outbreaks were likened to working in a war-zone with crisis management a key skillset. Staff workload increased and the enhanced level of reporting to multiple agencies through different routes requiring duplication of efforts was noted:

“There was an awful lot of paperwork involved with it, and a lot of it was repetitive paperwork. So for example, if you had staff off, so say you had three staff and then today I got a phone call, that’s four staff, then I have to admit those four staff again, so it was just almost like continual…the reporting of the same information again and again.” *(4, Manager & nurse, other adult home)*.

#### Staffing

Staffing has been challenging throughout. Individuals displayed flexibility around roles with senior managers required to replace domestic or catering staff, those no longer in frontline roles resuming care, and significant uptake of additional hours:

“We were working from the minute we got up in the morning till the minute we went to bed. For about six weeks, nobody had a day off. It was… Yes, it was pretty horrendous” *(11, Manager & nurse, older adult home)*.

Whole-home testing could often result in workforce depletion. Participants reflected on the contingencies to respond, with support from their own companies (if part of a group), use of agency staff and local staff-banks. Local staffing resources were often unable to respond in a crisis. Those in rural areas commented on specific staffing challenges due to the links between high community prevalence of infection and their staffing (e.g. outbreak at local school impacting staff). Initial support planned in the summer of 2020 became unavailable, even during significant pressures associated with later outbreaks in 2022. Agency staffing was usually considered only as a last resort, due to the increased risks of working in multiple locations. Some areas offered NHS staff as a resource however these were often not available or there were challenges around the roles staff were asked to fulfil:

“I’m a senior nursing auxiliary, I’m a staff nurse, I’m a nurse specialist and I don’t do that because that’s not in my job description. They just could not see past the job description, that was the sad thing.” *(15, Agency nurse, older adult home)*.

Several of those interviewed described ‘moving-in’ to stay in the home to cover additional duties.

Staff were mindful of the need to occupy residents, but restrictions made this difficult. The ability to adapt was often dependent on staffing levels. Some reported enhanced use of digital tools and technology (including online quizzes, supporting residents to use social media, enabling video calls with family members, reminiscence activity using online/digital resources and personalised playlists on iPods for each resident) with an emphasis on adapting for resident capabilities and preferences. Others created activity boxes to keep residents stimulated and occupied in their rooms and increased one-to-one activities including nail care. Homes for younger adults reported a tailored approach, often facilitated by a greater staffing resource.

#### Adaption to remote professionals & families

Staff also had to adapt to facilitate assessments by remote health professionals. This resulted in additional workload and responsibilities and variation in the extent to which staff felt comfortable in extending their role:

“Using the example of SALT, I can assist somebody – of course I can recognise if they’re choking or whatever – but I don’t have the skills to make the decisions right, how much thickener am I going to put in somebody’s food or fluid? Or what level, is it puréed, is it semi-soft? We were gauging that having had a ’phone call or a Teams meeting with somebody….But that level of responsibility almost became ours throughout the pandemic” *(13, Manager & nurse, older adult home)*.

They noted lost opportunities for shared professional development and learning because of specialists working remotely. Maintaining communication with relatives during restrictions required adaptation. Staff used digital tools and technologies to share photos, stories and videos of residents with their loved ones. Managers and senior staff committed time to provide weekly calls or emails to compensate for the lack of in-person contact.

#### Balancing harms & managing risk

The pandemic brought new harms for staff to balance, for example weight loss due to lack of social dining versus the risk of contracting the virus. This, more visible and measurable change, was one where there was confidence adapting practice to bring people together or to involve family members to minimise recognisable harms.

Those caring for people living with dementia, reflected specific challenges in their understanding of risk, the need to protect them and others and the extent to which guidance recognised the needs of this vulnerable group.

Risk assessment was seen as a positive tool to individualise for specific contexts and resident needs:

“we can always risk assess and put things in place or people to go out in the car because it is based on their mental health. I think that helped. I think that was maybe three months or four months into the pandemic, there was a shift about saying you can’t go out. Where actually if you have certain behavioural things…. you know you can because this is for your mental health.” *(7, Senior management & AHP, other adult home)*.

Others acknowledged that these approaches relied on adequate knowledge of the risks, which became more apparent and changed after vaccination. Those caring for younger people felt guidance was overly restrictive, compared to their peers elsewhere in the community.

Overall, it was clear that experience and confidence were needed to work outside of guidance and that this was weighed against the risk of external criticism:

“We made the very brave decision to put masks in place before they were made mandatory. It almost felt like we were having to keep it a secret that we had done that, almost like we were doing something wrong that was against the guidance. But we were just trying to protect our staff…. Of course, it come to fruition that masks were needed at all times” *(9, Senior management & nurse, both types of home)*.

### Emerging inequalities influencing residents’ care

#### Access to healthcare

Many participants described the impact of receiving correspondence from Primary Care advising that residents would not be able to access hospital care, including for non-COVID illnesses. They were often asked to distribute letters to families. They described widespread issuing of Do-Not-Attempt-Cardio-Pulmonary-Resuscitation (DNACPR) forms for all residents, irrespective of age and health status. Two of those interviewed also described receiving ‘verification of expected death’ paperwork for all residents. While accepting that hospital care and resuscitation were not appropriate for all, many residents already had anticipatory care plans and DNACPR forms pre-pandemic, these blanket approaches met visceral opposition from staff who advocated to individualise:

“I thought, oh my goodness, is this going out across Scotland, is every single care home resident now being pushed aside and basically this blanket policy of no treatment. And I thought, we surely have not sunk that far so quickly within days of this all kicking off, that we’ve come to this. I was shocked, absolutely shocked, and I thought, okay, but we need to do our best here, we need to do our absolute best.” *(26, Senior management & nurse, older adult homes)*.

Many refused to send on the information, as they thought it would cause distress. Others sent it and contacted families to agree a shared plan. Staff recognised the limits of care which they could provide within their home and valued the opportunity to escalate where they felt additional input may be. Although comfortable with providing supportive care if they felt the situation was futile, the blanket inability to escalate care was a disruption to their professional practice and left them feeling isolated and unsupported.

Several reported that efforts to access specialist care were met with hostility and refusal to attend from a range of practitioners. These responses were associated with the presence of COVID-19 within the home or with professional judgements about the appropriate place of care. Those working in homes without on-site nursing staff felt uncomfortable diagnosing dying without oversight from medical staff and found window and telephone visits unsatisfactory:

“Like someone was like physically dying they would give you a phone consultation, that’s not right, someone is dying, like you need to come and physically see them with your eyes instead of taking my word for it.” *(33, Senior carer, older adult home)*.

Staff expended time and emotional energy advocating for residents’ health:

“But I just feel as if I was advocating for the residents, and I was fighting with the GP surgery probably on a daily basis to get people to come and see the residents. And you just think, do you know what, we’re all in this boat together but it was very much…it became them and us very, very quickly.” *(6, Manager & nurse, older adult home)*.

This contrasted with those who received continuous or enhanced support from their primary/community care teams including care for residents in an outbreak.

#### Access to resources

Staff in homes with early outbreaks noted patterns in resident deterioration resulting in reduced intake plus varied effects of desaturation and skin changes. Individuals sought help to try to ameliorate these, e.g., by sourcing subcutaneous fluids, oxygen. However, structural barriers were encountered, only overcome by individual effort and persistence:

“I was begging the doctors for subcutaneous fluids for them, just to stop them becoming dehydrated in the hope that that would make their symptoms less as well….I think we could have been much better prepared for that to say, well look, here’s what COVID does, it makes you sick, it causes you to be dehydrated, so the first thing you’re going to do if somebody’s not eating and drinking is you’re going to get fluids and you’re going to put them up, to give them a fighting chance.” *(37, Manager & nurse, older adult home)*.

Managed outbreaks had rapid access to testing infrastructure and direct access to results. This contrasted with the lack of access initially. Some were able to access tests by sending staff to collect and deliver them at their regional hospital, but for many, requests for testing were denied or restricted. Participants with early outbreaks reflected that this resulted in missed opportunities to control outbreaks due to the prevalence of asymptomatic/presymptomatic presentations and for some no diagnosis at all, despite a presentation, deterioration and death consistent with COVID-19 disease:

“we had full PPE and everything else but, sadly, that didn’t stop the spread in March, towards the end of March…. And the staff were a bit distraught because they’re saying, we’re following all the guidance, we are changing PPE, we wear masks, how can this be? But there was a lot of not known at the start, so the incubation time or the period of the virus spreading, that was too late then, and that wasn’t the staff’s fault because their residents could have perhaps been together before there was the distancing and everything else that came in.” *(29, Senior management & nurse, older adult home)*.

#### Managing external risks of infection

Staff had to manage the risks associated with new or returning residents arriving from hospital. In the early stages, testing before discharge was not routine. In several instances the COVID-status of individuals was not communicated before transfer:

“…the GP then said, yes, but you know she’s positive, don’t you, she’s COVID positive and the nurse was like, no, we didn’t. She’d been back with us, I think, about 24 hours by that point and I’m thinking, how many staff had been working with her during this time? Now she was nursing in her bed but still, there was people in and out of that room ’cause she needed a lot of care and I’m thinking, oh my goodness.” *(26, Senior management & nurse, older adult homes)*.

This placed residents and staff at risk and, in some cases, was thought to have initiated an outbreak within the home. After national guidance about testing before hospital discharge was instituted, adherence was reported as inconsistent. Participants were sympathetic towards the pressures NHS teams faced but emphasised the need for effective handovers and testing before discharge to facilitate collective safety and continuity of care.

### Tension between staff experience and evolving external approaches

**Fig. 2:**
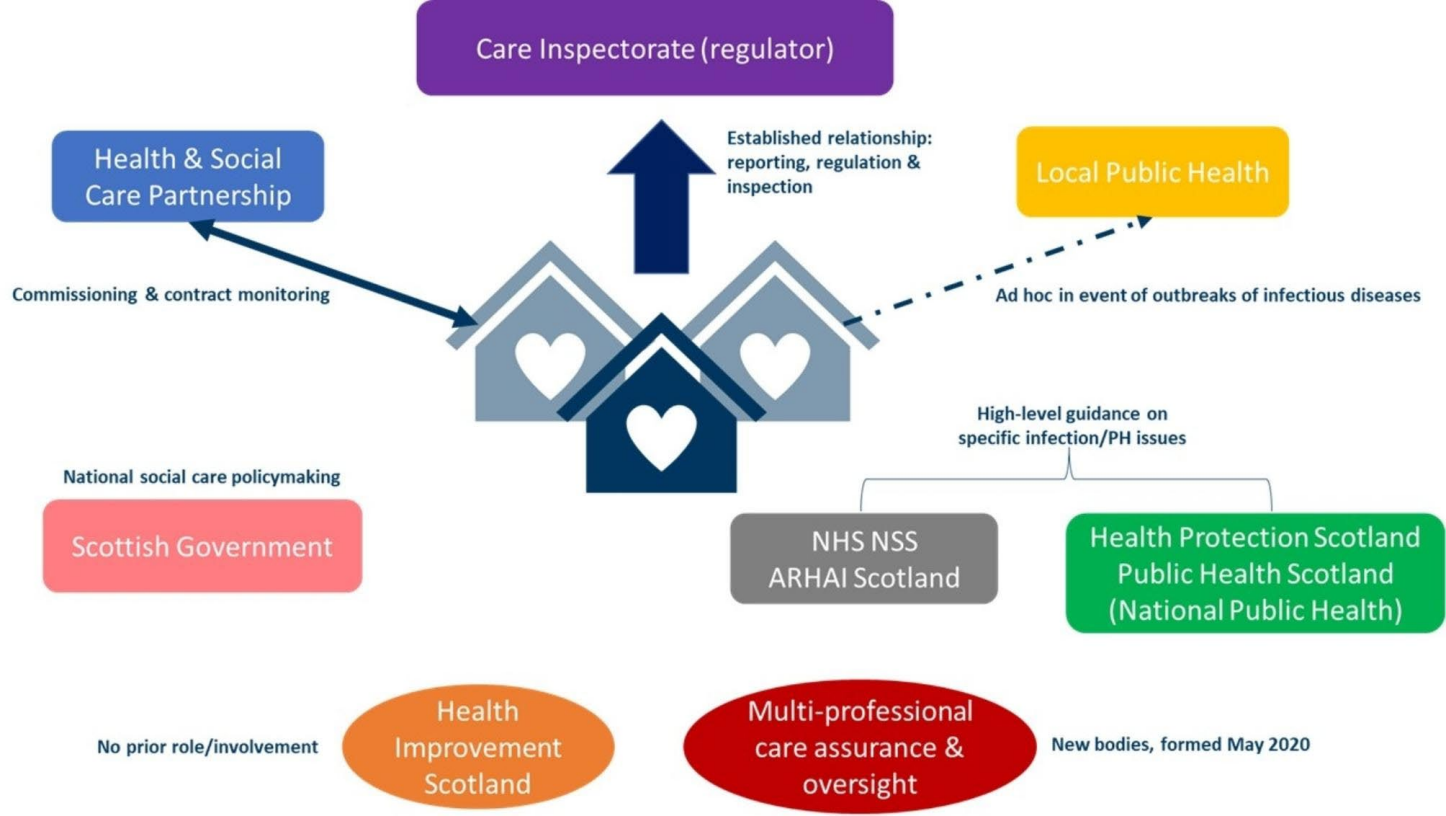
Relationships of national bodies to care homes before March 2020 – all of which were involved by summer/autumn 2020.NHS National Services Scotland (NSS) Antimicrobial Resistance and Healthcare Associated Infection (ARHAI) Scotland Health Protection Scotland became part of Public Health Scotland in Spring 2020

Figure 2 maps the organisations which from summer 2020 all had had frequent and direct involvement in care homes, charting their relationships before the pandemic.

There was a recognition that the regulator had adapted their practices to work remotely, using their adapted risk assessment tool. However, this resulted in variation around visibility, frequency of contact, change of personnel and inspections:

“We saw a change in the Care Inspectorate from being an organisation that I respected and I felt supported us to an extent but equally challenged us, to one that had lost their way….They took this puritanical view of things having to be perfect in an imperfect world when we were still learning, they were still learning, the world was still learning.” *(24, Senior management & nurse, older adult homes)*.

Participants questioned the timing of inspections and assurance visits, which often took place after homes reopened following outbreaks, on the day of re-opening when staff returned after sick leave. There was a sense that retrospective judgment on homes was unhelpful, and disappointment that input was not sustained in a time of crisis:

“I don’t mean to be quite judgemental or anything but I think Care Inspectorate and social workers kind of disappeared. I thought a pandemic you can’t get to see people that they would be the first people out to put eyes on people to make…are you safe? Is everything all right?” *(7, Senior management & AHP, other adult home)*.

Missed opportunities to provide practical support on incorporating new practices (e.g. donning/doffing PPE) before, or during an outbreak were highlighted, rather than reacting after a serious outbreak and identifying improvements. Support from those working remotely was repeatedly criticised, particularly where their advice conflicted with experiences on-the-ground and where practical help was needed:

“I just think that if people that were working from home could have been in and helping in the care homes with the right information and helping them before going in and criticising people at a very difficult time. Because let’s face it, nobody in this world knew what was going on.” *(25, Manager & nurse, older adult home)*.

Staff were challenged by changes they were asked to make to reduce the risk of infection, with variation between organisations in what was advised/required. Many felt that advice to ‘clinicalise’ the environment contradicted established practice and worsened the impact on residents:

“this is people’s homes and for years we were…for decades we were driven away to move from it being institutions and, you know, at points you were told to take people’s soft toys away and their soft furnishings were an infection control hazard and, you know, it… They were telling us to strip people’s rooms back to that cell like environment while they were stuck in it. It was awful.” *(1, Senior management & nurse, older adult homes)*.

The way in which change was requested through inspections and audit implied criticism, compounded by a lack of communication about changes expected before visits:

“it was taking away from the home environment as well, and the care home was made to feel quite clinical. And it was something like they noted on the audit, so it was like…it was almost as if it was a practice or an observation that it wasn’t good enough” *(16, Manager, older adult homes)*.

“you are assessing us against a framework that we don’t have and that is the problem. You have not given us the guidance beforehand to say this is what we are going to be looking at, this is what we are doing. You are measuring us against something that we don’t know.” *(8, Manager & nurse, older adult homes)*.

The purpose, role and need for the comprehensive, system-wide assurance and oversight teams was questioned:

“For us it felt like a bit of a kick in the teeth. We were never approached in any way to say, look, how are you managing? What are you doing? It was just a case of, right, we need to send out the NHS to you because you are just a care home.” *(23, Manager & owner, older adult home)*.

Some participants felt that this change implied deficiencies within care homes, rather than in the wider health and social care response to the pandemic, and that this was unhelpful in fostering constructive relationships. The governance of the new system and relationship between the Assurance Teams and the Regulator was a frequent source of discussion. There was recognition that many involved had no experiences of working in care homes and that this had created tensions, not recognising the specialism of care home practice. This included staff’s passion to uphold residents’ rights to autonomy balanced against maintaining collective safety in a communal environment:

“our residents make their choices about how they live. It is not like somebody being in a ward for seven days and we are basically saying, that is your bed and this is whatever. Our people are making choices.” *(8, Manager & nurse, older adult homes)*.

Others felt there was a positive contribution to be offered highlighting their role in sharing good practice and connecting services.

### Psychological impact

Abandonment was a common emotion in the initial pandemic response, particularly for homes which experienced significant outbreaks:

“We were just really let down at the time. The way I feel is like I felt everybody’s life was in my hands. I feel that normally as a nurse. People’s lives are in your hands but you have always got that support of people to ask.” *(5, Deputy manager & nurse, older adult home)*.

Participants spoke of the powerlessness they felt, being unable to stop residents from dying, despite following guidance. These impacts were felt more keenly where relationships had been close and enduring:

“We’ve been caring for some of these ladies for ten years. One of the residents that had passed away, I started my job the same week she moved in, so it was like we were constantly together….I saw her five days a week, every single week, unless I was on annual leave. Like how do you not grow a bond like that?” *(33, Senior carer, older adult home)*.

Staff and residents were reminded of those they had lost as they started to come together again.

COVID-19 posed a significant risk to staff as well as to residents and this had a bearing on their mental health with the sense of ‘putting your life on the line’ ever-present:

“one of my nurses thought she was going to die. She was, you know, standing in front of a very poorly man and her words to me were, I was standing here thinking, we’re all going to die here. ….It still upsets me to this day because it’s, like, how frightened must she have been and never took a day off. Never…you know, came in, they came in on days off, everything.” *(22, Manager, older adult home)*.

Participants recognised that not all their colleagues had been able to cope with those fears, generally responding with compassion to those who had been unable to work in outbreaks.

Some participants described interactions with visiting professionals or inspection teams who told them that they felt practice within the home contributed-to or caused resident deaths:

“And one point the consultant…we were talking about infection control, and I said, well, you know, we absolutely did our best with infection control measures, and he went yeah, but your residents died.” *(21, Manager & nurse, older adult home)*.

All those who experienced this described profound emotional impacts of these statements. The ongoing Crown Office Investigation was raised as a particular source of negative psychological impacts for staff, relationships with families and uncertainty (Table 2).

**Table 2:** Experience of involvement in Crown Office Investigation

**Established in May 2020 and required reporting of all care home onset deaths from COVID-19 deaths until December 2022 for investigation by Police Scotland**
**Work to collate submission**: “how in-depth and how time-consuming and how harrowing it actually was….” *(11, Manager & nurse, older adult home)*
**Nature of information collected**: “why are we being investigated? None of these questions ask, did you spend time with the resident? Did you give them comfort? What did you do? It is not, it is wanting to know the layout of the rooms, where their window is, what medications they were on. It is wanting to see food and fluid charts. It doesn’t want to know that you stimulated them or that you actually spent any time with them.” *(5, Deputy manager & nurse, older adult home)*
**Impact on relationships**: “I felt it made our families maybe question… Or everybody’s family in a care home question, god, have care homes put people at risk and have they… Is it their fault that people have died…… You know, you’re breaking bad news to say that somebody had died, which is the worst thing to do ever, and then you need to try and give them the heads up that by the way you might get the funeral director calling you, but you also might get the police phoning you. Try not to be alarmed.” *(1, Senior management and nurse, multiple older adult homes)*
**Impact on staff: “**The impact on staff’s mental health because of that. The fear that you have forgotten to write a temperature down or record your name accurately somewhere or you have not had this training or that or whatever it might be….You were then worried about everything you had documented and whether it was done right and did you make the right decision.” *(9, Senior management & nurse, multiple homes, both older and other adults)*
“I think it’s been destructive. I think it’s been hurtful for a lot of staff. Some have left the profession and sector because of it. It is not proportionate.” *(24, Senior management & nurse, multiple older adult homes)*
**Juxtaposition of end-of-life-care experience and police investigation**: “it was the disconnect between all that. And I was wanting to say to the police, I really, really care, my staff really, really care, how can I get that through to you that I know I’ve done nothing criminal. I actually know that, I know my staff haven’t either and I know, I’ve seen the tears, the sweat, the toil, and yeah, having to actually get this on paper to say, do you not know how much we cared and care, I don’t know.” *(26, Senior management & nurse, multiple older adult homes)*
**Uncertainty in outcome**: “….we have had no feedback from that. We have had no acknowledgement they have even received the paperwork. We have had nothing. We submitted there, I think the first submission was late 2020, first death was April 2020, and we have had nothing from them….There is no progress report, there is no end date.” *(8, Manager & nurse, multiple older adult homes)*
“And I think that hangs over everybody. I don’t like using this saying but it’s like a noose on your neck, not knowing what’s going to come of it.” *(29, Senior management & nurse, older adult home)*
**Global challenge of managing COVID-19 before vaccination**: “And I just don’t even understand it, I don’t know where that’s coming from because if hospitals couldn’t keep people alive, how did they think we were going to manage? If hundreds of thousands of people have died across the world, why would these care homes in Scotland be able to keep them alive? They’re not going to. And I think that seems to have got lost somewhere.” *(10, Senior management & nurse, multiple older adult homes)*

The lasting impacts have been wide ranging in terms of job satisfaction and morale, contributing to loss of staff:

“I think this used to be the best job in the world and I think being able to make a difference, just lead a team to make a difference, whether it be big, massive or a wee toaty, toaty…but to be able to go home every night knowing that you’ve made a difference to somebody…And see now, I struggle to come to work…… And I hate the fact that COVID’s made that change” *(6, Manager & nurse, older adult home)*.

However, there was a recurring sense that some of what staff experienced went beyond the pandemic and was reflective of views of their occupation, with a desire for wider professional and public respect:

“a lot of people kind of just see care assistants as care assistants….you’re just a care assistant, you are just a drop-out from school sort of thing. So, I think they treat us like crap a lot of the time. Not everyone obviously. I don’t know what they could do to make it better. Maybe just treat us like human beings instead of dirt on the bottom of their shoe” *(33, Senior carer, older adult home)*.

### Compassionate leadership & teamwork

Compassionate leadership and teamwork were evident in the ways staff responded to the challenges faced.

Participants described adapting information provision and training, noting differences in learning styles among colleagues (e.g. visual learners). They described sharing rapid updates, using a range of approaches including newsletters, leaflets, posters, online alerts, emails, texts, and WhatsApp messaging. These were supplemented by increased rapid team meetings/huddles to keep people up-to-date and forums to discuss longer-term change. Efforts were made to make training and learning fun, through activities and competitions:

“Training-wise there is e-learning we have got an e-learning platform. In order to get an uptake of staff for that we run wee competitions for three staff to get such and such module done get a prize. The best reflection gets a prize. We do stuff like that just to make it a wee bit more fun. It is working.” *(5, Deputy manager & nurse, older adult home)*.

Participants reported adapting guidance from national bodies due to the volume, frequency, timing of dissemination and format:

“I suppose that’s my job is to kind of cut the wheat from the chaff and pass on the nuggets of information that people need to know rather than…’cause you can’t give people all that information and expect them to work it out themselves, do you know?” *(35, Manager & nurse, older adult home)*.

The lack of accessible resources and easy-read versions was highlighted as a significant limitation. Homes with greater organisational infrastructure and administrative support often designated individuals to manage this. All acknowledged this as a significant burdensome task, particularly when change was required immediately without time to implement.

Key to managing change was involving staff and providing rationale for change:

“making sure everyone is aware of what’s going on, and giving them a chance to speak, and putting their ideas forward. So, everyone was involved in any changes that we made within the building, working practices, everyone was involved in that.” *(32, Deputy manager & nurse, older adult home)*.

“I would just kind of talk to them all the time about ‘we tried this and this works’. But I’m always open to ideas anyway so I think it’s really important for staff to feel included…. So I’d say, right, the guidance today is this, you know, and I think it’s because of this and I think this is why they want us to do it this way. And make them feel there’s a rational reason.” *(10, Senior management & nurse, older adult homes)*.

For one participant, the opportunity to involve all parties in change was critical, with visiting changes coproduced:

“because we are very relationship focused so any changes in the guidance was put out to families. Put out to everybody connected to our service, what is your view? What do you want? So that we could make decisions that were informed by the people that live and work in our care homes. I think that rescued us at times. And sometimes that threw up a bit of an anomaly because when the guidance changed that families could come in, I was overjoyed. I thought, yes, finally. I assumed all our families would be equally overjoyed, but it wasn’t the case. There was a real 50/50 split.” *(9, Senior management & nurse, both types of home)*.

Debriefing and reflection were helpful tools to maximise learning from outbreaks. Managers and more senior staff commented on their management style being accessible, present, and supportive. Flattening hierarchies, authentic leadership and flexibility in roles was important, particularly when working during outbreaks but maintained thereafter.

Participants consistently expressed willingness to share their learning with others. When offered opportunities to engage these were welcomed, including sharing experiences of controlling outbreaks and involvement in national policy changes around testing pathways, visiting and, digital developments:

“even in a crisis, we don’t just need to drown here, we can thrive, we can actually get better in what we do and more than survive it, but actually bring some good to all this terrible stuff that’s going on.” *(26, Senior management & nurse, older adult homes)*.

There was a collective sense of the need for reflection and learning:

“so as long as there’s a positive outcome and going forward a learning….our generation hasn’t had a pandemic before so you didn’t fully know what to expect and no one knew the pressures that were going to hit us or the outcomes. It was learning as you went.” *(18, Deputy manager & nurse, older adult homes)*.

This attitude acknowledged the unique challenges of managing a pandemic and mistakes which resulted across the whole system, but a sense of focusing on the learning to ensure such mistakes are not repeated in future.

Looking across the data as a whole and reflecting the diversity of experiences shared, Table 3 summarises effective sources of support participants received. These are broadly classified as: interpersonal, professional networks, developmental and structural. These form an important part of transferable learning to inform future supportive responses.

**Table 3:** Effective sources of support identified by participants

	Source	Evidence
**INTERPERSONAL**	**Accessible professional advice and support, provision of personal contacts for queries**	“the communication has been great, the emailing. I feel on first name terms with them all at public health” *(5, Deputy manager & nurse)* “so helpful and so knowledgeable. If they weren’t able to tell you, they got back to you and they were so realistic about things.” *(8, Manager & nurse)*
	**Check-in calls from trusted professional with prior understanding of service**	“they were phoning us I think about once a week, sometimes twice a week in the beginning, which was really nice, and I think it really helped bridge that gap between us, because it’s always, oh god, the Care Inspectorate, nobody wants them coming to your door, but actually, they were very supportive and realistic about things” *(17, Manager)*
	**Continuity of care from dedicated GP practice with contact and visits maintained when requested**	“Doctor (*redacted*) supported us 100 per cent and was here when we needed her, both for staff and residents. Excellent cover we get” *(25, Manager & nurse)* “our GP practice has been very supportive throughout the whole pandemic. Anything we’ve needed, it’s a phone call away, they continued to visit throughout the pandemic, even during outbreaks here. There was a real support there from them” *(16, Manager)* “we have one linked person, so they were very good in coming to help us” *(31, Manager & nurse)*
	**Dedicated professional contact line**	“after a wee while, we did get another number, which was straight through the NHS-24 line. That was a really great service because you got the phone answered very quickly and you would just take advice from them” *(27, Manager & nurse)*
	**Support from families**	“We have such a good rapport with the relatives as well. It is their appreciation. They used to message and they were so grateful for looking after their loved ones when they weren’t allowed in.” *(5, Deputy manager & nurse)* “our families would champion us all the time. They would send gifts to staff, chocolates, send cakes in to the home. They would send cards in just saying how grateful they were. We have got private Facebook groups just for families and they would say to the staff continuously what an amazing job you are doing.” *(9, Senior management & nurse)*
**PROFESSIONAL NETWORKS**	**New or enhanced support networks with other homes or with professionals, without differentiation by ownership**	“think it was just going onto the meetings and something, like I just said about the not knowing, there was 16 other managers there not knowing either. So it was sharing, and then sharing of practice, things that have worked well in the other homes, things that didn’t work so well, scrutiny visits, feedback from other homes, so you can prepare for the next…like when they were coming to you.” *(16, Manager)* “no longer are we (*redacted*) separate homes, we’re a team of different homes from different companies that now share everything… if you find something good and it works, tell the whole world. Share it, let everybody use it.” *(13, Manager & nurse)*
	**Peer support within organisation**	“So yeah, so I just put a call out, you know, has anyone else had this issue then I’d generally get some help from a colleague, but it’s very much, kind of, peer-on-peer support” *(21, Manager)*
	**Scottish Care webinars & organisational advocacy**	“But I think through Scottish Care, I believe that’s been a lifeline for me. Any surgeries that they held, because they really did understand and the people in the call were all in the same boat. And I firmly believe that they helped me as a professional through because they’re chairing these meetings and the chat that went on, and knowing that you had somebody to reach out to” *(29, Senior management & nurse)*
DEVELOPMENTAL	Formal leadership development training (e.g. My Home Life, Queen’s Nursing Institute)	“I am a My Home Life convert, I use those tools all the time in my working life….I have been using those tools to converse with people for a long time in terms of how I speak with people” *(8, Manager & nurse)* “I was fortunate enough to have completed the Queen’s Nurse programme, and so the journey I went through with them, I was able to pass on to my team” *(29, Senior management & nurse)*
	**Improved access to training, education & professional development opportunities**	“We get every week information about what is happening locally, staff training that even I have tapped into that I would have never been able to get into if it wasn’t for COVID” *(7, Senior management & AHP)* “they’ve offered training for the staff, and we have accessed it …it’s been quite good and beneficial for staff, they’ve quite enjoyed it.” *(4, Manager & nurse)*
	**Sharing learning with new partners**	“We opened our arms to them and said, come in and we will teach you. Actually we made strong partnerships with all the teams that we have been part of that team and teaching them the ways of care homes….So there has been lots of learning and shared learning” *(9, Senior management & nurse)* “…actually showed and demonstrated all our skills to each professional that came through and helped them building their confidence” *(29, Senior management & nurse)*
**STRUCTURAL**	**Access to Personal Protective Equipment via Hubs**	“when we had an issue with supply, as in the PPE stuff coming in from the suppliers…there was a huge shortage because – well the shops were the same – and the deliveries weren’t getting through and the QA team here immediately sent me to the hub – I mean, we’ve replaced them – but we just got the supplies, got them on that day and started them immediately. So that was very supportive” *(13, Manager & nurse)*
	**Financial support for additional costs**	“We didn’t have staff uniforms, for example. So I spoke to the sustainability fund people and they had said, well, if you need to buy uniforms for your staff and you wouldn’t have normally done it if it wasn’t for the pandemic then you can claim for it.” *(23, Manager & owner)*
	**Testing infrastructure which enabled care homes to test and access results**	“it did become much easier after a while to get through in terms of the results would then go through straight to yourselves, we got setup with an NHS email accounts and stuff like that. So I could pick up the results instantly for any mass testing” *(37, Manager & nurse)* “the testing was all freely available at that point so it was easy enough. We just phoned up Place Name and they would send us a form to fill in and tests were delivered next day and picked up the same day” *(23, Manager & owner)*

## Discussion

### Findings in context

Our study sought to address an evidence gap, listening to the experiences of care home staff in a range of roles and settings, to identify learning from the pandemic and beyond. Identifying the impacts on staff of managing changed relationships, advocating for residents in the face of inequalities and managing the psychological impacts, we also heard about an adaptability to respond and compassionate responses in a crisis.

Our findings align with other recent international research which focused on aspects of the pandemic response in-depth including the impact of visiting restrictions, [33] the challenges for those supporting people living with dementia [34] and responses to new infection prevention & control measures within a care home setting [35, 36]. The negative impact on care home relationships has also been described including the impact of stress and burnout among staff [37]. Another UK-based study focused on the impact on nursing staff, highlighting the experience of moral distress as a consequence of feeling unprepared [9]. A Canadian study of nursing staff focused more on the compassionate leadership skills and professional development to support staff [11]. The challenges of delivering high-quality relationship-centred palliative and end-of-life care have also been recognised in care homes internationally [38, 39]. Other aspects, such as the timing of issuing changes to guidance and challenges around regulation and oversight have been highlighted in Scotland [40, 41]. The interdependencies between care homes and their local and national systems have been recognised alongside the need for expertise in care home managers to navigate these systems [42].

### Strengths & limitations

We focussed on staff working directly within homes with outbreaks and were successful in recruiting a sample of professionals working across different settings, geographies and supporting different client groups. Our participants tended to have years of experience in the adult social care sector and managerial-level staff are over-represented. This under-represents care staff and those of shorter duration of service – both common in the UK care home workforce [43]. Furthermore, although our participants reflected on the experiences and impact of residents, we are limited by not hearing their voices directly and the voices of those who support and care for them. The lack of research directly involving residents has been a significant limitation in the UK pandemic response, reflective of wider challenges in governance and infrastructure for social care research [44].

Undertaking the analysis as part of a small team with varied professional and personal experiences during the pandemic provided opportunities for discussion and challenging of assumptions made based on those wider experiences. We could provide support for each other in managing the emotive and ethically challenging experiences shared by participants. Working with our Steering Committee of professionals provided further rigor to our approach as we presented conclusions and questions arising from the analysed data and worked with them to ensure theme naming had face validity among key stakeholders.

### Implications for practice & policy

In addition to acknowledging the profound psychological effects of managing COVID-19 on care home staff and the extent to which they adapted and advocated for their residents, there are important implications of our findings. Eight arising questions have been identified as priorities to construct a more proportionate response for the care home sector, drawing on questions or challenges raised by participants (Table 4).

**Table 4:** Arising questions for reflection & exploration

In common with many countries, the devastating effects of the COVID pandemic on those living and working in care homes in Scotland were not anticipated or prepared-for.
To ensure future responses are cognisant of the contexts, vulnerabilities but diversity of the care home population, these questions merit consideration to construct a proportionate response:
1. **Where were the Health & Social Care standards in the pandemic response and why were other principles and values prioritised for so long?**
2. **How can we improve interprofessional communication at transitions of care, in particular, at hospital discharge?**
3. **How could the community, primary and palliative care response to care homes be consistently strengthened to support homes caring for residents during outbreaks?**
4. **How can we ensure there a staffing contingency with the attitudes, skills and behaviours to support care home residents when staffing is depleted in an outbreak?**
5. **What impact has the Crown Office investigation had on the management of the pandemic in care homes and the sector as a whole?**
6. **What is the purpose of inspection in an outbreak situation and when is the optimal timing of this to support residents?**
7. **How do we improve the accessibility of guidance and enable services time to respond and implement change?**
8. **How can we better identify, evaluate and embed learning and evidence generated from care home practice to develop the evidence-base for decision-making? **

These include reflection on the rights and principles used to inform the response. The Health and Social Care Standards Principles (Dignity and respect, compassion, be included, responsive care and support and wellbeing) [45] were not always evident in practice and policy-making. Although emergency situations necessitate directive approaches, upholding rights and values around individualising and personalising care must remain a priority even in a crisis. For example, the harms from blanket-DNACPR issuing need to be acknowledged in terms of impact for individuals and to redress lasting negative effects on in public understanding of this important component of anticipatory care planning [46, 47].

In March 2020 guidance was issued which recommended that individuals with COVID-19 should be managed within the care home, with in-reach services provided [48]. However, much focus of the initial response was on acute care, with those in the community not having the same access to care or enhanced support. Our participants provided examples where access to hospital care was denied for care home residents. Given wider policy and practice preferences to deliver care closer to home, [49] investment must be made to enable enhanced community and primary care responses, including hospital at home services to support the healthcare needs of care home residents, [50] in addition to equitable access to acute care when required. These aspects are included in a new framework to enable consistent support for the healthcare needs of residents [51]. The NHS England Framework for Enhanced Health in Care Homes [52] has not been adopted in Scotland.

We welcome the Independent review of inspection, scrutiny and regulation of social care support [53] which is underway in addressing some of the challenges participants described on the roles of organisations and bodies to enable a coherent and proportionate response.

Our study found evidence of compassionate leadership within the sector which deserves recognition. Other UK survey research has recognised the commitment of those who have remained part of the care home workforce [8]. The need to value and enhance the leadership which exists within the care home sector itself has been recognised internationally as a priority to avoid future harm [54].

In addition, prioritising identification of areas of uncertainty from care home practitioners to mobilise timely collaborative research to address them is an ongoing priority to ensure there is effective generation of research for application in practice [55, 56].

The pandemic has exposed neglected challenges in the provision of long-term care nationally and internationally [57–59]. Scotland is in a period of potentially radical reform with the proposed creation of a National Care Service [60]. Although the scope is broad, key lessons include understanding the diversity and specialist nature of care home practice and the intrinsic qualities and skills within the workforce which are often unrecognised. There is a need to ensure that the positive contribution which care homes and their staff can make in supporting those with complex needs is included in the public and professional narrative, to sustain the sector [61].

## Conclusions

Global knowledge of managing COVID-19 in care homes has evolved and the risks associated have been radically reduced with vaccination. However, there remains valuable learning from those with lived experiences given the role the care home sector plays in supporting adults with complex needs. We found:

1. The diversity, professionalism and expertise across the care home sector has been under-recognised and under-utilised; involvement needs to be embedded.
2. Effective sources of support were underpinned by respectful relationships and continuity, tailored to individual contexts.
3. Further analysis is needed on the evolving regulatory, oversight and assurance context including how to restore confidence and autonomy within the sector.
4. Directive, blanket approaches to the care home population countered established principles of care home professionals practice, requiring individual advocacy to challenge.
5. There is a collective need to engage in whole-system reflection, learning (not blame) and reconciliation to avoid future harm.

### Electronic supplementary material

Below is the link to the electronic supplementary material.

Supplementary Material 1

## Data Availability

The data generated and analysed in this study are qualitative interview transcripts which contain sensitive data which could identify research participants. In accordance with the ethical approval for the study granted by the University of Glasgow, they will not be made publicly available.

## List of abbreviations

UK: United Kingdom
ENRICH Scotland: Enabling Research in Care Homes, Scotland
NHS: National Health Service
CPAG: Clinical and Professional Advisory Group for Adult Social Care
