# Supplementary material for: Listening and learning: a qualitative study of Scottish care home staff experiences of managing COVID-19 between March 2020-August 2022
Source: BMC Geriatr. 2023 Sep 7;23:544. doi: 10.1186/s12877-023-04251-z (PMC10485989; doi:10.1186/s12877-023-04251-z)
Supplement: Supplementary file 1 — Supplementary Material 1 [file 12877_2023_4251_MOESM1_ESM.docx]

**Supplementary materials**

| **Supplementary file 1:** | Summary of key policy & guidance changes in Scotland | 2 |
| --- | --- | --- |
| **Supplementary file 2:** | Semi-structured interview guide | 3 |
| **Supplementary file 3:** | Summary of themes with additional illustrative participant quotations | 4 |
|  |  |  |
|  |  |  |

**Supplementary file 1**

**Box: Summary of key policy & guidance changes in Scotland**

| **Care home clinical guidance:** produced and issued in March and May 2020  **13^th^ March 2020**: including social distancing, isolation, reducing visitors, managing COVID-19 within the care home, <https://www.careinspectorate.com/images/COVID-19_-_Letter_from_Cabinet_Secretary_for_Health_and_Sport_-_Social_care_guidance_-_13_March_2020.pdf>  **16th March 2020**: including presentation of illness, anticipatory care planning, admission and transfer guidance <https://scottishcare.org/wp-content/uploads/2020/03/COVID-19-ClinicalGuidanceforNursingHomeandResidentialCareResidents-Final26March.pdf>  **15th May 2020**: including PPE advice, admissions and testing, healthcare support fir residents, wellbeing and mutual support  <https://www.webarchive.org.uk/wayback/archive/20200516095432/https:/www.gov.scot/publications/coronavirus-covid-19-clinical-and-practice-guidance-for-adult-care-homes/>  **Testing before hospital discharge:** individuals leaving hospital going in to care homes have been required to have negative COVID-19 tests from 21^st^ April to present. Initially two negative polymerase chain reaction (PCR) tests were required. <https://www.hps.scot.nhs.uk/media/1925/covid-19-information-and-guidance-for-care-homes-v10.pdf> This has subsequently been reduced to one lateral flow test.  **Enhanced professional clinical and care oversight teams:** Each Health Board and Health and Social Care Partnership were required to introduce a multidisciplinary professional clinical and care oversight teams from 17^th^ May 2020. Their remit was resident care, infection prevention and control, staffing and testing in outbreaks/ongoing surveillance. <https://www.careinspectorate.com/images/documents/coronavirus/Coronavirus28COVID1929-enhancedprofessionalclinicalandcareoversightofcarehomes2800329.pdf>  **Personal Protective Equipment:** Responding to challenges experienced by social care providers and unpaid carers obtaining adequate supplies of PPE, PPE hubs were established in April 2020 to help secure supplies (<https://scottishcare.org/wp-content/uploads/2020/10/PPE-Hubs-MoU-v2.0-final.pdf>)  **Funding**: Additional financial support for social care providers was first introduced in December 2020 and included resources for staffing, personal protective equipment and administrative costs. Staff support ended in March 2023 (<https://www.gov.scot/publications/coronavirus-covid-19-financial-support-arrangements-for-social-care-providers/documents/>)  **Crown Office and Procurator Fiscal Service investigation into COVID-19 deaths**: established on 13^th^ Mary 2020 by Lord Advocate. Requiring all deaths of those deemed to have care-home onset of COVID-19 infection to December 2022 be reported and investigated by Police Scotland in Operation Koper.  **Vaccination programme**: In line with the UK Joint Committee on Vaccination and Immunisation advice, care home residents and staff in older adult care homes were prioritised for COVID-19 vaccination roll-out. The national vaccination programme commenced on 14^th^ December 2020 and has continued to be delivered by visiting teams of vaccinators. Vaccination has remained voluntary for residents and staff. |
| --- |

**Supplementary file 2: Semi-structured interview guide**

**Listening and learning: the experiences of care home staff in Scotland in managing COVID-19**

**Semi-structured interview guide**

Opening (pre-recording)

- Confirm individual has read participant information sheet and signed consent form electronically
- Remind them that their responses will be kept confidential and neither they nor the care home they work in will be identified in any results
- That they are free to stop the interview at any time if they wish

Interview

If happy to go ahead, turn on dictaphone and say, before we move into the discussion part of the interview, I have a few structured questions to ask which we are asking everyone who takes part. Can you tell me:

**What your role is in the home:** *sub-categorise based on response*

**How many years you have been working in adult social care**: less than one/one to two/three to five/six to ten/ten to fifteen/sixteen to twenty/more than twenty

**What is your working pattern**: full time/part time

**During the pandemic did you work in**: one care home service/ more than one care home service/care home and NHS service *[if more than one home, for rest of questions focus on main place of work]*

**What is the broad grouping of the care home you work in**: older adult service/other adult service/both

**What is the broad geography of the care home you work in**: urban/rural/both

**How many beds does the care home you work in have**: less than 30 beds; 31 to 60 beds; 61 to 90 beds; more than 90 beds

Thank you, as you know the aim of this study is to explore the experiences of care home staff in Scotland of managing COVID-19 within their homes to help inform understanding and future practice, so can I start by asking you about your experiences………….

Thereafter, can you tell me about………….

- Identifying COVID-19 infection among residents
- Your experience managing outbreaks
- Your impression of the impact of outbreaks on residents
- Reflections on the external support received or required
- Experiences of the interface with healthcare, specifically GPs and the acute sector
- Experience of hospital discharge during the pandemic
- Reflections on outbreak investigation and scrutiny/oversight/assurance
- How to sustain learning from outbreaks
- What has helped in supporting resilience?
- Any other reflections on how learning should inform future practice?

I’ve come to the end of my questions, is there anything that we haven’t discussed that is important to you to share?

**Thank you for taking time to share your experiences**

**Supplementary file 3: Summary of themes with additional illustrative participant quotations**

| **Theme** | **Illustrative quotations** |
| --- | --- |
| **Fundamental change to care home relationships**  *Challenges for new residents*  *Respite & day services*  *End of life care* | “You don’t realise how much the lounge area was such a communal area and everybody knew everybody and all the families spoke to each other and every family knew every resident…..Everybody was just like a big family…… We’ve still not got that sense of community back and I think that’s really missed in the home.” *(6, Manager & nurse, older adult home)*  “she used to go up and down the corridor in her wheelchair speaking to everybody. She died a couple of months later, not COVID-related. But again broken hearted because she thought everybody had deserted her. And there was lots of that.” *(22, Manager, older adult home)*  “you’ve seen some horror stories of people shouting through letter boxes and demanding to get in. We would never ever be in that situation and that’s a horror story that happened, and it was horrific to see…..that is just a practice that I would never expect to see or hear about in a care home setting” *(29, Senior management & nurse, older adult home)*  “I went to the other side of her room, so I was about four metres away and I took my mask off so she could actually see my face, and she was just like, it is so nice to just see a face in here. It's like, that's mad, you've been here for like nearly three years and you've never seen our faces. It must be scary for them, they must be terrified.” *(33, Senior carer, older adult home)*  “it’s a huge thing, they need the support, it’s the social interaction, coming in, it’s massively important for our residents. It’s their friends, it’s people they’ve lived with, as I said, we’re a really small area, you know, and it’s getting the gossip” *(32, Deputy manager & nurse, older adult home)*  “things must have been really at crisis for you to be willing to take a respite place to bring that husband, wife, son, daughter to the door of the care home and not be able to come in and not be able to come and see the rooms like you used to…..I can't imagine how incredibly difficult that was for families” *(9, Senior management & nurse, both types of home)*  “it was so very quick, very fast. And I think you just…I think when someone’s palliative, a usual kind of palliative death, you have that time, you know, to make them comfortable and you know that the family’s there, all that kind of thing, you know, you’ve put all that in place and you do what you can to enable a really good death. And with this, you couldn’t really do any of that.” *(10, Senior management & nurse, older adult homes)* |
| **Adaptation in uncertainty**  *Staffing*  *Adaption to remote professionals*  *Balancing harms and managing risks* | “as a nurse you’re trained to have a diagnosis, and this is how you manage it but the fact we didn’t have any evidence. There was no…there was no research to tell us what we should do” *(1, Senior management & nurse, older adult homes)*  “we changed from seven hour shifts to 12 hour shifts. I knocked up a two-week rota within about half an hour and they were instantly up for it. It was a combination of us trying to manage it and staff understanding what we were trying to do, and it was seamless” *(23, Manager & owner, older adult home)*  “I didn’t end up having to use agency staff. I just rolled up my sleeves and be there on the floor, and encouraged the staff to step up as well, and volunteer to do extra to help out.” (31, Manager & nurse, older adult home)  “they were on a laptop, and we had to go and undress the wound. We had to go and measure the wound, we had to assess the wound. We had to do the paperwork and then send it to them for them to then give us the prescription of what they were to do.” *(8, Manager & nurse, older adult homes)*  “let’s be honest, sometimes you’re the only nurses in the care home and all the decisions are coming down to you. So they do need to have that, and there is, there’s a huge degree of autonomy and you’ve just got to hope that you do the right thing at the right time.” *(27, Manager & nurse, older adult homes)*  “They just became so frail and they changed in their eating, they didn’t want to eat. You have only got so many staff, so we even changed the meals. Rather than those all going out at the same time we did them in stages. We did them in groups of five so that the residents could sit with staff to eat” *(5, Deputy manager & nurse, older adult home)*  “I think we lost sight of people, it was about keeping people safe and not their personhood, but you’re not safe if you’re not…..when you don’t have all these other elements of life, but then you’re not safe if you’re dead. It is that balance, I think we’ve all had a lot more experience of how to get that balance right now” *(30, Advanced Nurse Practitioner, older adult home)*  “Yeah, and you can’t do that with people with dementia. There’s no understanding that actually people need people, especially if you’re distressed or you are confused. You don’t know what’s going on. People have abandoned you. That’s how you feel. Because my family can’t come and see me, but oh they could look at me through a window but how can I touch them or feel them, or oh I’m not allowed to do that, you know. I just think that’s, you know, that is, it’s not human, is it? It’s just not.” *(11, Manager & nurse, older adult home)*  “what we’re doing now, when you’re speaking to infection control saying, this is how I’m managing it. Yes, there’s a risk, but it’s worth taking, do that from the start next time…..I could totally understand these 50 bedded homes having visitors wandering in and out is just not doable, but we could do it here to a certain extent, but we weren’t allowed to” *(17, Manager, older adult home)*  “We just wrote risk assessments to our hearts content for every single person that we felt needed to see somebody or needed out their room…..Anybody that we felt needed a visitor, this was before the essential visiting, we put a risk assessment in to say that they actually did need to see their visitor. It worked in our favour because thankfully we didn’t lose anybody through getting COVID that we allowed people in when they weren’t meant to come in.” *(5, Deputy manager & nurse, older adult home)* |
| **Emerging inequalities**  *Access to healthcare*  *Access to resources* | “I decided not to issue that to the families and agreed with all the managers that, no, we’ll deal with every single case as an individual and we’ll fight for what we can if it’s going to help them” *(1, Senior management & nurse, older adult homes)*  “you’ve got some people who it was not COVID-related and you think the escalation, they still deserved to get an assessment and treatment at hospital as much as anyone does” *(18, Deputy manager & nurse, older adult homes)*  “he was looking at him through his bedroom window, to do his…for us to be able to give him end of life care and all the stuff to…so just in case medication, all that…because, you know, we just wanted to be clear what we were doing” *(22, Manager, older adult home)*  “We requested COVID testing from the GP, and we were told no, and that there was no mandate to test people in the community.” *(21, Manager & nurse, older adult home)*  “we saw testing coming in for various…certainly in hospitals and yet we had nothing. We had nothing for our staff. We had nothing for our residents. So initially when we lost residents at the beginning it was suspected COVID because we had no way of actually knowing.” *(24, Senior management & nurse, older adult homes)* |
| **Tensions between staff experience and evolving external approaches**  *Introduction of oversight teams*  *Care home practice* | “at the time I think the biggest help would have been for these…you know, for our regulators, the Care Inspectorate, or any trained professional to just pull on a tunic and could come in and help us……would have been really nice but instead they were phoning us all day. we had…..everybody and their granny on the phone……wanting to ask what was going on and what were we doing about it, when really, we were actually trying to care for people and nurse them through this” *(1, Senior management & nurse, older adult homes)*  “you’ve done your visits from the past two years from an office, we’re here at the frontline. We know what we need to do, we know how to do it, and we always put our residents first.” *(37, Manager & nurse, older adult home)*  “We were actually here on the ground doing the actual day to day reality of working within that environment, and whilst we had people who were saying, we will support you, we are here for you, it got to the stage where we felt that what they were doing was criticising” *(4, Manager & nurse, other adult home)*  “I think initially there was resistance to the NHS coming in because it was portrayed the wrong way. It was started as this assurance and oversight and they are coming in to teach us and various things. I think where that has been unsuccessful is where they have kept that type of a model. We already have the Care Inspectorate so do we need the Care Inspectorate or have we got these teams, which are we having?” (*(9, Senior management & nurse, both types of home)*  “Can you ever imagine, ever in your wildest dream, me being sent into the hospital to check to oversee if they are doing things right. It just wouldn't happen. There would be a complete outcry. But it is actually okay for a colleague that did their nursing maybe with me to come out and oversee my work. We talk about partnership working but there needs to be an equality there and it is just absolutely not there. The things that have been done to care homes would never be done to the NHS, never. It wouldn't even be considered because there would be an absolute public outcry.” *(9, Senior management & nurse, both types of home)*  “The ethos of the service is to support residents to live the lives they want to live” (14, Senior management, other adult home)  “I think when it’s communal living as well, the way a care home is, you have such a responsibility for other people in your vicinity as well as just your general responsibility to others. Which I think, it does take away sometimes people’s ability to make more independent decisions” *(30, Advanced Nurse Practitioner, older adult home)* |
| **Psychological impact**  *Relationships with residents*  *Wider perceptions of social care sector* | “I think a lot of people wondered that, is it because older people are just dispensable? That was almost how you felt. It was almost like I am saying I have never felt so undervalued in my whole career. It was almost like the people I have worked with and I have learned from my whole career you really felt the stigma of they don’t matter, they don’t matter. We really were left. We were left by everybody. You were making huge decisions.” *(9, Senior management & nurse, both types of home)*  “we were watching people that we truly loved and cared for fade away to the point of death in front of our eyes. The frustration of a motivated, caring team to be put in that situation, it was shocking.” *(3, Senior carer, older adult home)*  “your morale, almost demoralisation, again the grief they were going through because they’d lost a lot of residents. When you’re getting everyone out the lounge and into their own areas and you’re looking about and you’re thinking, this are all the residents we’ve got, the place is half-empty. That kind of thought. When they were in their rooms, it was more hidden but then when they came down into the lounge, it exposes the whole home and there’s all these empty seats” *(18, Deputy manager & nurse, older adult homes)*  “we had a lot of agency staff and the NHS staff were coming in, so people’s names and photos would be on the door. And then so sometimes one of those staff who didn’t know that person had died, would go into the room. And so they kind of complained that, oh, you should really have something on the door to say that this person isn’t here anymore…..So they put this little poster of an empty room. But it was so busy all the time, you didn’t actually really register a little thing like that. But the girls who came back to work, they found that heart-breaking because they came into work, they came into their shift, they’d walk along the corridor and there would be empty room, empty room, empty room, empty room. And they found that really difficult.” *(10, Senior management & nurse, older adult homes)*  “I think in an ideal world for me would be just for NHS nurses, any staff, to be treated on the same level as the care sector and not to have that where they think that the nurses aren’t as skilled because they are. They might be skilled in different things” *(5, Deputy manager & nurse, older adult home)* |
| **Compassionate leadership & teamwork**  *Guidance*  *Involving staff in changes*  *Debriefing and reflective practice*  *Leadership style*  *Sharing learning with others and involvement in change* | “We would do wee spot checks and quizzes, almost like who knows the order of this, who knows the order of that, what’s the common signs for COVID, what’s your atypical signs. And just kind of quizzed them a wee bit, like you’re talking about it every day so that it was that they knew…and they did all know but when you get quizzed by strangers and people in different uniforms, they kind of freeze. … It was very informal but as seriously as it had to be, but informal so it wasn’t too stressful for anyone.” *(18, Deputy manager & nurse, older adult homes)*  “So, it was the quantity of information, the quality of the information, I mean, if something changes, I would get the same information from about seven sources…..so I was just bombarded with information and changes to, for example, the PPE, that kind of thing and sometimes things would change in a day.” *(21, Manager & nurse, older adult home)*  “definitely could have been provided some information that is clearer, easier to read and understand” *(34, Manager, other adult home)*  “making sure everyone is aware of what’s going on, and giving them a chance to speak, and putting their ideas forward. So, everyone was involved in any changes that we made within the building, working practices, everyone was involved in that.” *(32, Deputy manager & nurse, older adult home)*  “I would just kind of talk to them all the time about ‘we tried this and this works’. But I’m always open to ideas anyway so I think it’s really important for staff to feel included…. So I’d say, right, the guidance today is this, you know, and I think it’s because of this and I think this is why they want us to do it this way. And make them feel there’s a rational reason.” *(10, Senior management & nurse, older adult homes)*  “it’s alright to look back and learn or think we could have done things better. That’s not a sign of weakness, indeed it’s a sign of strength.” *(24, Senior management & nurse, older adult homes)*  “to prevent the next bad episode we tried to prepare some contingency plan or have some debrief and prevent that for future and then update our business continuity plan” *(36, Manager, older adult homes)*  “I put on my nurse’s uniform again. And the two waves, those early days, I worked with my staff, mainly because I wanted to show, well if I’m asking you to care for these people and we know COVID’s around now, then I’m asking you to be at risk here, I’m going to be here alongside you.” *(26, Senior management & nurse, older adult homes)*  “I think just being yourself and actually getting your hands dirty and showing that you’re able just to get on with it with everybody else. And that’s probably the main thing. Because staff look to you as, if you do it, then are you expecting them to do it as well.” *(12, Manager & nurse, both types of home)*  “So, the whole hierarchy of being a team leader and having different levels within the team, that disappeared to some extent because I felt….the whole team needed to know that information because if anything happened to me, then they needed that information to be able to continue to work. And basically, that’s continued to this day.” *(11, Manager & nurse, older adult home)*  “And it didn't matter what your title was, we were all there to pull our weight and do whatever was needed in an emergency.” *(15, Agency nurse, older adult home)*  “we were one of the first care homes in Scotland to have positive cases of COVID……I feel that it was a privilege to be able to help and support the learning of other places through their COVID journey.” *(11, Manager & nurse, older adult home)*  “If we want to move forward, we have to focus on the strengths and rather than just continuously focusing on the negative aspects of it. We need to think about but what did we do well and how could we do a whole lot more of that the next time? Of course, we are learning from our mistakes.” *(9, Senior management & nurse, both types of home)* |
